# Effects of the Consumption of Low-Fat Cooked Ham with Reduced Salt Enriched with Antioxidants on the Improvement of Cardiovascular Health: A Randomized Clinical Trial

**DOI:** 10.3390/nu13051480

**Published:** 2021-04-27

**Authors:** Desirée Victoria-Montesinos, Raúl Arcusa, Ana María García-Muñoz, Silvia Pérez-Piñero, Maravillas Sánchez-Macarro, Antonio Avellaneda, Francisco Javier López-Román

**Affiliations:** 1 Health Sciences Department, Campus de los Jerónimos, Universidad Católica San Antonio de Murcia (UCAM), 30107 Murcia, Spain; dvictoria@ucam.edu (D.V.-M.); rarcusa@ucam.edu (R.A.); amgarcia13@ucam.edu (A.M.G.-M.); sperez2@ucam.edu (S.P.-P.); msanchez4@ucam.edu (M.S.-M.); antonio.avellanedagoicuria@elpozo.com (A.A.); 2 R&D Department, ElPozo Alimentación S.A., Alhama de Murcia, 30840 Murcia, Spain; 3 Primary Care Research Group, Biomedical Research Institute of Murcia (IMIB-Arrixaca), 30120 Murcia, Spain

**Keywords:** antioxidants, oxidized LDL, cooked ham, catechins, hydroxytyrosol, chlorogenic acid, malondialdehyde, cardiovascular risk, ABPM

## Abstract

The aim of the study was to analyze how cardiovascular risk factors can be modified using nutritionally improved cooked ham enriched with a pool of antioxidants to influence relevant metabolic targets. Sixty-five untreated subjects (49.2% males, 50.8% females, mean age 40.92 ± 9.03 years) with total cholesterol level ≥180 mg/dL or LDL cholesterol ≥130 mg/dL participated in a 8-weeks randomized, double-blind controlled trial. Participant in the intervention group (51.5% males, 48.5% females, mean age 41.6 ± 9.8 years and mean BMI 25.1 ± 3.6 kg/m^2^) consumed cooked ham enriched with antioxidants (100 g/d) and controls (49.9% males, 53.1% females, mean age 40.2 ± 8.3 years and mean BMI 26.3 ± 3.2 kg/m^2^) received placebo. At 8 weeks, oxidized LDL decreased significantly between experimental and placebo groups (*p* < 0.036). Experimental group differences were also significant (*p* < 0.05). Similar findings in malondialdehyde, total cholesterol, high-sensitivity C-reactive protein, and interleukin 6 were observed in the intervention group. Significant between-group differences in these variables were also found, except for total cholesterol and interleukin 6. The effects on inflammation and oxidation support the direct action of these antioxidants on the etiopathogenic factors of atheromatous plaque. We also observed an improvement in the lipid profiles among the subjects.

## 1. Introduction

In recent years, the association between diet and cardiovascular disease (CVD) has been extensively evaluated and investigated [1,2,3]. Atherosclerosis is the primary cause of CVD [4], and is an excessive inflammatory and fibroproliferative response, which becomes chronic and does not exert a protective effect against a series of attacks on the arterial intima that favor the deposition of lipids, which influences the progression of atheroma plaque [5]. Plaques grow with proliferation of fibrous tissues and surrounding smooth muscle and bulge within the arteries and consequently reduce blood flow. The connective tissue produced by fibroblasts and calcium deposits lead to hardening of the arteries. The uneven surface that eventually forms in these blood vessels causes flow obstruction due to clot formation and thrombosis [6]. The chronic, excessive, and unbalanced caloric intake, high in glucose [7] and fat [8], can generate a state of obesity and a greater production of white adipose tissue, which promotes the secretion of pro-inflammatory factors, thus producing oxidative stress [9,10]. According to the Global Burden of Diseases study published in 2016, conducted in 51 countries between 1990 and 2016, diet is responsible for more than 9.1 million deaths from CVD [11]. Diet-related CVD mortality is higher in men than in women, with the risk being higher in older age groups [12]. Oxidized LDL (ox-LDL) and the ox-LDL/low density lipoprotein cholesterol (LDL-C) and ox-LDL/total cholesterol (TC) ratios are the markers most commonly studied as CVD risk factors [13]. ox-LDL comes from the oxidation of lipids and apoproteins present in LDL-C, which leads to a conformational modification that more easily promotes the atherosclerotic process [14,15]. The ox-LDL stimulates endothelial cells to produce proinflammatory molecules that recruit monocytes and promote their differentiation to macrophages [16]. The activation of these cells and foam cells leads to the production and release of proinflammatory molecules such as interleukin beta-1 (IL-β1), interleukin-8 (IL-8), tumor necrosis factor-alfa (TNF-α), and interferon-γ. Activated macrophages can produce and release reactive oxygen species (ROS), which increase the oxidation of LDL-C and the amount of ox-LDL in the intima [17,18]. IL-6 is a proinflammatory adipokine commonly generated in overweight and obese people since adipose tissues produce such adipokines [19]. Plasma MDA is a strong marker of lipid peroxidation and oxidative stress, and high levels of plasma MDA can lead to an increase in cardiovascular risk (CVR) [20].

There are many epidemiological studies that associate the Mediterranean dietary pattern with a lower CVR and mortality, as well as lower levels of inflammation [21,22]. The most recent meta-analyses show a clear association between foods with high antioxidant contents and a decrease in CVR due to changes related to risk factors [23]. Serrano et al. [24] previously carried out a study in rats using cooked ham enriched with dietary phenolics, observing an improvement in body composition, oxidative stress biomarkers, and inflammation-related biomarkers. The same cooked ham enriched with antioxidants was also used in this clinical trial.

The most recent meta-analyses show a clear association between foods with high contents of polyphenols and a decrease in CVR due to changes related to risk factors, such as a decrease in total cholesterol or blood pressure [23,25]. Furthermore, various biochemical markers of dyslipidemia, inflammation, oxidative stress, and insulin resistance are among the modifiable cardiovascular risk markers that respond to dietary interventions [26,27,28].

Some antioxidants, such as catechins, hydroxytyrosol, and chlorogenic acids, generate benefits at the cardiovascular level through their impact on the bioavailability of nitric oxide (NO), endothelial function, lipid profiles, and blood pressure [29,30]. However, the amounts of antioxidants ingested through food intake are insufficient for achieving a prevention–dose relationship, even if dietary recommendations are followed [31,32]. The intake of foods enriched with antioxidants can help to achieve this prevention–dose relationship and reduce the risk of CVD.

According to the latest reports issued by the Organization for Economic Cooperation and Development (OECD) and the Food and Agriculture Organization of the United Nations (FAO), the intake of meat and meat products at the international level is on the rise. Meats and meat products comprise 23% of the total Spanish market, representing the most frequently consumed foods [33,34].

Several articles show that the consumption of red meat, especially processed meat, is considered a cardiovascular risk factor [35,36,37]. However, there are inconsistencies in comparing the occurrence of these diseases in different populations. This is due, among other causes, to the foods habitually consumed by each of these populations. In most of them, most of the energy consumed comes from saturated fats, refined starches, sugar, high salt intake, etc. [38,39]. The cardiovascular risk generated by the consumption of processed red meat is attributed, among other things, to the high amount of saturated fats and sodium that this type of product usually contains [39,40,41]. In this study, a low-fat cooked ham with extremely low saturated fat and reduced salt content was used in order to reduce the possible risk that the matrix could have due to the content of these nutrients.

Using these meat products as a matrix containing a pool of antioxidants could help the population to meet the requirements for reducing cardiovascular risk.

The objective of this clinical trial was to analyze how cardiovascular risk factors (CRFs) can be modified using nutritionally improved low-fat cooked ham with reduced salt enriched with a pool of antioxidants (chlorogenic acids, catechins and epicatechins, hydroxytyrosol, zinc, selenium, and vitamin C) to influence relevant metabolic targets and examine the effectiveness of this ham on serum levels ox-LDL, cholesterol, triglycerides, and high-density and low-density lipoprotein cholesterol (HDL-C, LDL-C).

## 2. Materials and Methods

### 2.1. Trial Design

This study consisted of a randomized, controlled, double-blind, and unicentric clinical trial of two parallel branches according to the product consumed (experimental and placebo), lasting 8 weeks.

The study protocol was approved by the Institutional Review Committee of the San Antonio Catholic University (Murcia, Spain; CE031902) and was conducted according to the Declaration of Helsinki. The trial was registered at www.clinicaltrials.gov (identifier No. NCT04506749) in August 2020. The study was carried out at the Department of Exercise Physiology of the Catholic University San Antonio of Murcia (UCAM). The protocol was carefully explained to the subjects by the investigators, and then the subjects who decided to participate signed informed consent. Each subject was assigned a code in order of arrival. Subsequently, a researcher from outside the research group, through a software generator (Epidat v4.1 Epidat, Galicia, Spain), assigned each subject to one of the groups. Neither the researchers nor the subjects knew to which group the subjects belonged.

### 2.2. Participants

The studied group contained 72 participants of both sexes. The subjects had to fulfill all the chosen inclusion criteria (age between 30 and 75 years old, both sexes, Caucasian race, body mass index (BMI) between 20 and 32 kg/m^2^, and levels of serum LDL-C equal to or higher than 110 mg/dL or total serum cholesterol equal to or higher than 180 mg/dL under fasting conditions without pharmacological treatment). The participants also had to avoid all of the exclusion criteria: thyroid dysfunction, infection, chronic diseases, an ischemic–vascular event in recent months, treatment with drugs or nutraceuticals for hypertension, treatment with diabetes or hyperlipemia (statins) drugs that required monitoring plasma levels (digoxin, acenocoumarol, or warfarin), carrying out or intending to carry out any type of diet during the study, allergies or poor tolerance to any component of the product under investigation, having donated blood in the last month, having had major surgery in the last 3 months, being vegetarian, having ingested omega-3 or omega-6 supplements in recent months, treatment with niacin or fibrates, abusive alcohol consumption, seeking to modify nicotine habits, and participation in another clinical trial in the three months prior to the study.

### 2.3. Supplementation Protocol

The product under investigation and the placebo were provided by a local meat company. Both products had identical characteristics, with total fat contents below 1.5% and saturated fat and sodium below 0.5% (BienStar^®^, ElPozo Alimentación, Alhama de Murcia, Murcia, Spain). Both products presented the same appearance and featured identical organoleptic characteristics. As shown in Table 1, both products had similar compositions except in their antioxidant and polyphenol contents. The experimental product contained higher amounts of selenium, zinc, vitamin C, chlorogenic acids, hydroxytyrosol, catechins, epicatechins, and other phenolic acids than the control product. It is also low in saturated fat and reduced in salt. The presentation took the form of slices of cooked ham that were differentiated only by a code. After the completion of the study, it was revealed to which code each product belonged. The administration was oral, at 100 g/day, for 8 weeks with no protocol or daily intake pattern. As the product was given to the subjects every 15 days, the subjects were instructed to return the empty packages every 15 days, as well as at the end of the study, to check compliance with the supplementation protocol. For this purpose, the investigators retained a file with the dates, quantities, and batch codes of the products delivered and collected.

### 2.4. Study Settings

The subjects attended the laboratory several times. Twenty days before the start of the study, a recruitment phase was carried out, where potential participants were selected, informing them of the development of the study. Fifteen days before the intervention, preliminary visits (V0) were performed to select the subjects, during which a detailed anamnesis of the subjects was performed, including personal and demographic data, alcohol consumption habits, smoking habits, and diet. Written informed consent was also obtained. Whether the participants met all the inclusion and none of the exclusion criteria was determined. The cholesterol values were determined by blood analysis, and the participants were randomly assigned to one of the treatment groups (EXP or PLA).

During the experimental phase of the intervention, 2 visits were made: the basal visit (V1) and the final visit (V2). In both visits, blood samples were obtained from each subject’s antecubital veins to assess the lipid profile and analyze the antioxidant defense, oxidative damage, and inflammation markers. BP was determined following the ESC/ESH criteria [42], and bioimpedance was performed to evaluate possible changes in body composition (weight, BMI, fat mass, and muscle mass). At V1, 15 blister packs of 100 g of the experimental or placebo product were delivered to each subject, and every 15 days, another 15 blister packs were delivered. At V2, the surplus product was collected, and a collection notebook was filled in, including any possible adverse effects.

### 2.5. Study Variables

All the variables were analyzed at the beginning and end of the study after the uninterrupted consumption of the product. The subjects were informed that, during the intervention, they could not initiate/modify any treatment or modify their dietary or physical activity habits, as such changes could affect the parameters under study. The participants were informed that any changes to the aforementioned variables should be communicated to the investigators.

#### 2.5.1. Blood Sample Measurements

The subjects were instructed to arrive after a 12 h fasting period and were only allowed water intake up to the three hours before the extraction. For smokers, the last cigarette had to be smoked at least one hour before the extraction. Moderate–high-intensity exercise could not be performed in the previous 24 h. Blood samples were collected from the antecubital vein in vacutainer tubes with gel and a clot activator (Becton Drive Franklin Lakes, NJ, USA). The tubes were centrifuged, and the samples were aliquoted in eppendorfs and stored frozen at −80 °C until analysis. The laboratory studies explored (a) the lipid profiles (TC, triglycerides (TG), LDL-C, and high-density lipoprotein cholesterol (HDL-C)) using a clinical chemistry analyzer (BA400 Biosystems) and ox-LDL using a Human Oxidized LDL ELISA kit (Catalog. No: E-EL-H0124 96T; Elabscience Biotechnology Inc.; Wuhan, Hubei, China) with a direct method; (b) inflammation-related biomarkers (hs-CRP via the colorimetric method using an ILAB 600 assay analyzer (Instrumentation Laboratory) and the levels of IL-6 with an IBL International High Sensitivity Interleukin-6 Kit (Interleukin-6 High Sensitivity ELISA REF. BE58061 96T; Hamburg, Germany)) via a direct method; (c) oxidative stress biomarkers (MDA using an ELISA kit from Elabscience (MDA (Malondialdehyde) ELISA Kit Catalog No: E-EL-0060 96T; Elabscience Biotechnology Inc.; Wuhan, Hubei, China)) with a direct method; and (d) antioxidant defenses (superoxide dismutase (SOD) using an ELISA kit from Elabscience (Human SOD1 (Superoxide Dismutase 1, Soluble) ELISA Kit; Catalog. No: E-EL-H1113 96T; Elabscience Biotechnology Inc.; Wuhan, Hubei, China) with an indirect method. The samples were kept frozen prior to measurement.

The assay procedure of the competitive ELISA was followed according to the manufacturer’s instructions. A serum pool was used as a quality control. Intra and inter-assay precision (CV%) was <10% for each determination analysed by ELISA kits.

#### 2.5.2. Blood Pressure Measurements

Blood pressure was taken using ambulatory blood pressure monitoring (ABPM), following the ESC/ESH criteria [42].

ABP was determined using a Holter Spacelabs Healthcare onTraK SL_90227 (Holter) at the baseline and end of the study. The cuff was placed on the subject´s nondominant arm so that the subject could continue to perform his or her daily activities without impediment. The Holter was placed at the waist by means of a belt, which allowed the Holter to continue measuring BP while the subject slept. The subjects were instructed to stop, not move their limbs, and remain silent each time the cuff was inflated. The Holter was programmed to measure once every hour for 24 h.

For each of the measurements made with the Holter, an average of 24 hours’ worth of data were obtained, including systolic blood pressure (SBP), diastolic blood pressure (DBP), and overall average BP. The average day- and nighttime BP were determined according to the time range, considering both day- and nighttime hours, and the SBP and DBP were obtained for each. Nighttime period was considered from 22:00 h to 6:00 h. In turn, the pulse pressure (PP) was determined according to the differences between the SBP and DBP. The relevance of PP lies in its capacity to estimate the elasticity of the arteries, giving it predictive value for cardiovascular disease risk [42]. For this variable, the 24 h average was measured, as well as the differentiation by time slot.

The dipping or circadian pattern of BP refers to a drop in blood pressure during the night compared to the day. For this variable, the percentages of the nocturnal decreases with respect to the diurnal average BP were considered and classified as follows: riser (a nighttime BP increase or no nighttime BP decrease with respect to the day), non-dipper (a 1–10% decrease in nighttime BP compared to daytime values), dipper (a 10–20% decrease), and extreme dipper (a decrease of more than 20%). Another variable, the tensional load (the systolic or diastolic limit percentage), is the percentage of readings from the Holter above 140/90 mmHg in the waking period and above 120/80 mmHg during the sleep period.

#### 2.5.3. Anthropometric Variables

Anthropometric variables were also included, such as bodyweight, BMI, fat mass, and free fat mass. These were determined via foot-to-foot bioimpedance (Tanita BC-420M, Tanita Corporation, Arlington Heights, IL, USA) [43,44].

#### 2.5.4. Physical Activity

Physical activity was assessed using the Global Physical Activity Questionnaire (GPAQ), which classifies subjects as little, moderately, or highly active [45,46].

#### 2.5.5. Dietary Habits

For the analysis of the dietary survey, the subjects recorded their food intake with a 24-h questionnaire on 3 nonconsecutive days including 2 workdays and 1 weekend day. The data were processed using computer software (Dietsource^®^v3.0).

### 2.6. Statistical Analysis

The sample size was calculated according to the ox-LDL as the main variable of the study. Considering a standard deviation of ox-LDL levels of 620 pg/mL [47], for a precision of 400 pg/mL with alpha risk of 5% and statistical power of 80%, 30 subjects in each group were needed, increasing to 36 subjects per group assuming a 20% loss to follow-up.

The per-protocol (PP) data set was analyzed, that is, all participants who completed the 8 weeks study period. Categorical variables are expressed as frequencies and percentages and continuous variables as mean and ± standard deviation (SD). Data analysis included the chi-square (χ^2^) test for comparison of categorical variables between the study groups at baseline or t-Student for comparisons of quantitative variables. Normality was tested by the Kolmogorov–Smirnoff test and homoscedasticity by the Levene test.

To analyze the differences between the groups in the evolution of the different variables, a parametric test such as an analysis of variance (ANOVA) for repeated measures was carried out, with time (baseline and final) as the within subject factor, and intervention (EXP and PLA) as between-subject factor (for the post hoc analysis, Bonferroni tests were carried out), or nonparametric test such as the Mann–Whitney test to compare the variation experienced by the variables during 8 weeks consumption.

Statistical significance was set at *p* < 0.05. The SPSS version 21.0 (IMB Corp., Armonk, NY, USA) was used for statistical analysis.

## 3. Results

**Figure 1:**
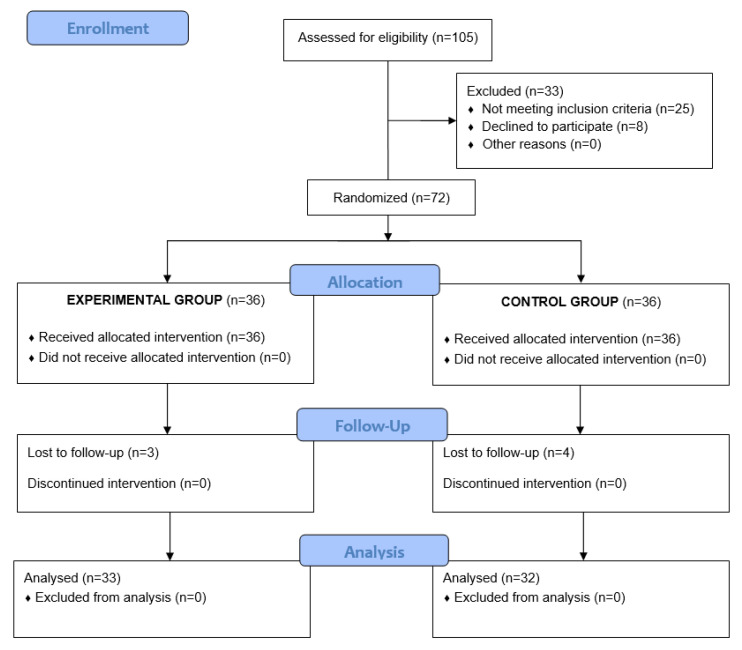
Flow chart.

A total of 105 subjects of both sexes were recruited for the study, 25 of which did not meet the eligibility criteria, and eight declined to participate. Therefore, 72 subjects were included in the study (Table 2 shows the demographic data) and subsequently divided into two groups (experimental group (EXP) and placebo group (PLA)), each containing 36 participants. After considering loss to follow-up, only 65 participants, 51.5% males, 48.5% females and the mean age of the study population was 40.92 ± 9.03 years, completed the intervention and were included in the final statistical analysis depicted in Figure 1. As shown in Table 2, both groups were homogenous at baseline in terms of parameters such as weight, BMI, fat mass, fat-free mass, TC, LDL-C, and BP.

The lipid profiles showed improvements in the monitored biomarkers at the end of the intervention after the consumption of the low-fat cooked ham with reduced salt enriched with antioxidants, particularly for serum ox-LDL, the comparison of baseline (443.3 ± 277.6) and final values (364.2 ± 212.9) showed statistically significant differences in the EXP group (*p* < 0.05), whereas differences were not significant in the PLA group. Between-group differences in LDL-ox were statistically significant (*p* < 0.036). When the subjects were stratified according to their BMI in two groups (BMI > 25 and BMI < 25), we can observe a correlation between a BMI > 25 and a decrease in oxLDL (*p* < 0.026). However, in the group with BMI < 25 there is no statistical significance in the decrease of oxLDL, although a downward trend is observed.

Serum total cholesterol was another parameter that improved after product consumption, significantly decreasing by 7.7 mg/dL (*p* < 0.032) in EXP group, while differences were not significant in the PLA group and between-group differences were not significant. No significant differences were found in the rest of the lipid profile variables. However, there is a positive correlation between the decrease in oxidized LDL and LDL (*r* = 0.632; *p <* 0.001).

Regarding the inflammation parameters, significant improvements were observed in the two biomarkers tested, hs-CRP and IL-6, characterized as related to the acute phase of inflammation. Both biomarkers decreased in the group that consumed the antioxidant-rich cooked ham. Serum Hs-CRP decreased by 0.66 mg/L (*p* < 0.006) in the EXP group, no significant differences in the PLA group. However, the values of IL-6 decreased significantly, by 0.67 pg/mL (*p* < 0.001), in the EXP group and increased by 0.19 (*p* = 0.443) in the PLA group. Comparing the evolution between the groups, no significant differences were observed for this variable (*p* = 0.331). The results seem to confirm an improvement of inflammation after product consumption.

The antioxidant profiles were evaluated based on oxidative damage and antioxidant defenses by comparing the mean MDA and SOD. Serum MDA decreased 37.1 ng/mL (*p* < 0.05) in EXP group, no significant differences in PLA group. When comparing the evolution between groups for this variable significant difference are observed (*p* = 0.035) which seems to affirm that the product under investigation protects against oxidative damage. Significant changes in the serum SOD were not recorded.

In relation to the values related to blood pressure, no significant differences were shown in any of the studied parameters for systolic, diastolic, and pulse pressure in terms of 24-h, daytime, and nighttime blood pressure. The same was true for the pressure load and nocturnal SBP and DBP (dipping), as well as the qualitative systolic and diastolic dipping (risers, non-dippers, dippers, and extreme dippers). Therefore, it cannot be said that the consumption of the product improved blood pressure.

According to the body composition data evaluated by bioimpedance, all the parameters remained constant during the intervention, so it could not be confirmed that the product affected body composition.

Physical activity habits were not modified. At the beginning of the study 46.2% of the subjects were classified as inactive, and 53.8%, as active. At the end, 49.2% were inactive, and 50.8% were active. The subjects also maintained their dietary habits, since after the nutritional analysis, no differences were found in macronutrient and energy intake between baseline and the end of the intervention.

The results for all the study variables at the baseline and the end of the intervention are reported in Table 3, Table 4 and Table 5 as means and standard deviations (mean ± SD).

## 4. Discussion

The present study was a double-blind, controlled, and placebo-controlled clinical trial carried out over 8 weeks to evaluate the effect of the daily consumption of 100 g of low-fat cooked ham with reduced salt enriched with vitamin C, zinc, selenium, and a combination of chlorogenic acids, catechins, and hydroxytyrosol on the lipid profiles, blood pressure, antioxidant status, inflammation, and anthropometric measurements of subjects. After the established time, the intake of cooked ham was associated with beneficial modifications of a series of factors related to CVD, such as lipid profiles, as well as biomarkers of antioxidant and anti-inflammatory status.

A significant decrease was observed in ox-LDL values, which is one of the most important findings of this study. Plasma lipoproteins are very precise biomarkers of oxidative stress in the arterial wall due to their interactions with endothelial cells and the ease with which their surface lipids are oxidized. These LDLs carry polyunsaturated fatty acids (PUFAs), target substrates in lipid peroxidation, which is why LDLs are among the molecules most strongly affected by oxidative stress. Ox-LDL promotes endothelial dysfunction and the formation of atherosclerotic plaque, so reducing ox-LDL levels could reduce cardiovascular risk [48,49]. When comparing our study with other clinical trials, we found mixed results. In a clinical trial in patients with metabolic syndrome, a 20% reduction in ox-LDL values was achieved compared to in the control group by using capsules containing 9.32 mg of hydroxytyrosol [50]. Perrone et al. [51] carried out a similar clinical trial, in which a 37.5% decrease in ox-LDL levels was observed after the consumption of 9.4 g of hydroxytyrosol. Another clinical trial carried out on a population of hypertensive patients without pharmacological treatment also showed significant results in the reduction of ox-LDL levels after the consumption of 6.64 mg/kg of phenolic compounds [52].

In vitro tests, such as those conducted by Gordon et al. [53], have shown the efficacy of chlorogenic acid in lowering ox-LDL. It was observed that an increase in chlorogenic acid produced an increase in the delay of LDL oxidation.

In our clinical trial, the cooked ham was enriched with 6.58 mg of catechins and epicatechin per 100 g of product. Catechins and epicatechin are polyphenols that have been extensively studied, especially for their presence in green tea. The efficacy of these antioxidants in combating oxidative stress has been observed, showing that these polyphenols are capable of reducing free radicals in addition to intervening in multiple metabolic pathways, although their mechanisms of action remain unclear [54]. One of the proposed mechanisms of action is that catechins seem to be linked to the regulation of endothelial NO synthase (eNOS), producing an increase in endothelial NO and, subsequently, endothelium-dependent vasodilation, which leads to a decrease in the inflammatory response and lipid peroxidation [55]. In a dietary intervention trial lasting 4 weeks, the efficacy and antioxidant power of cocoa were assessed in 60 subjects. After the intervention, there was a significant decrease in ox-LDL in the group that consumed 4 g of cocoa (220 mg of flavonoids) [56]. In another 4-week trial, it was observed that the consumption of 153.44 mg of epicatechin and 14.56 mg of catechins contained in cacao markedly decreased ox-LDL levels [57]. Kirch et al. [58] showed that the consumption of 25 mg of epicatechin in subjects with metabolic syndrome over 2 weeks did not result in a significant decrease in ox-LDL values.

Ultimately, the consumption of phenolic compounds produces an observable decrease in ox-LDL in subjects.

The product used in our clinical trial was enriched with vitamin C, selenium, and zinc. A clinical trial conducted by Van Hoydonck et al. assessed how the consumption of a vitamin C supplement (500 mg) affected a population of smokers. No significant differences were observed for the ox-LDL variable [59].

There was no significant decrease in ox-LDL levels in another clinical trial in which the subjects took capsules with 180 mg of vitamin C, 6 mg of zinc, and 50 μg of selenium [60].

As previously mentioned, the consumption of polyphenols can reduce oxidative damage. Our data reveal how the consumption of catechins and epicatechin generates a significant decrease in MDA, CRP, and IL-6 and, in turn, leads to an increase in SOD. MDA is one of the most widely used biomarkers for measuring oxidative damage, as it reflects the peroxidation of polyunsaturated lipids. Elevated levels of MDA are directly associated with increased cardiovascular risk [61]. The proinflammatory effects of this ROS dysregulation were shown to yield an increase in inflammatory cytokines, such as CRP and IL-6. An effect observed under higher IL-6 concentrations is an increased risk of myocardial infarction [62]. In response to the oxidative stress, the body increases antioxidant enzymes to combat the increase in ROS. However, when the number of free radicals is excessive, the production of these enzymes, such as SOD, is insufficient [63].

Other studies, such as the one carried out by Noronha et al. [64] on obese women who ingested 450 mg catechin capsules, also showed significant decreases in MDA and IL-6. Spadiene et al. [65] reported that a nutraceutical from *Camellia Sinensis* L. featuring catechins and other polyphenols produced a decrease in MDA and increase in SOD.

Not all clinical trials conducted with catechins have provided significant results. For example, a clinical trial by Basu et al. [66] showed a nonsignificant decrease in IL-6 levels in a group that ingested infused tea, and no significant results were observed for a decrease in CRP.

The new cooked ham used for this clinical trial was enriched with vitamin C, selenium, and zinc. Selenium can modulate free radicals and inhibit NF-κB, thereby decreasing oxidative damage and suppressing biomarkers such as MDA [67]. Vitamin C is also a strong antioxidant [68], and zinc is one of the most important trace elements in the body, not only because it is part of more than 300 enzymes but also because it is a key element in antioxidant responses [69]. There are multiple articles that highlight the antioxidant capacity of these three elements. A clinical trial carried out by Kamali et al. [67] showed a significant decrease in MDA levels in subjects who ingested 200 μg of selenium. On the other hand, another study showed no significant changes in MDA and SOD levels after the consumption of 250 and 500 mg of vitamin C, respectively [70]. In other study, it was observed that supplementation with zinc (50 mg/d) for 8 weeks produced a significant decrease in MDA levels compared to placebo [71].

The product tested in our clinical trial contained 22.5 mg of chlorogenic acid per 100 g of product. Chlorogenic acid is an ester made up of caffeic acid and quinic acid. Multiple functions are attributed to this acid, such as the improvement of carbohydrate and lipid metabolism due to its antioxidant activity and the prevention of lipid peroxidation induced by hydrogen peroxide [72]. Martínez-López et al. [73] observed a significant decrease in MDA after the consumption of 74.2 mg/g of chlorogenic acid.

The cooked ham used in this clinical trial, which was enriched with a pool of antioxidants (chlorogenic acids, catechins and epicatechins, hydroxytyrosol, vitamin C, selenium, and zinc), contained several active compounds intended to attack different targets of the atherosclerotic process. Several studies have used combinations of antioxidants such as vitamin C, vitamin E, selenium, zinc, β-carotenes, and N-acetylcysteine to observe their effects on the prevention of cardiovascular diseases. Most of these studies showed that supplementation with a group of antioxidants reduced oxidative stress and inflammation in subjects [74,75,76,77,78,79,80,81].

A relationship between plasma metabolites and the effects of active antioxidant compounds has not yet been established. One area of future study could be the distribution of polyphenols at the tissue level to determine the different metabolic targets that the polyphenols act upon. There is currently very little information available on this topic [82,83,84,85,86].

It must be considering the limitations of the study, including the reduced sample size, the exploratory nature of the trial and the short treatment period. It would be appropriate to test the intake of the low-fat cooked ham with reduced salt enriched with antioxidants in long term.

Based on the main findings of this study, we conclude that the consumption of cooked ham that is low in fat and salt and enriched with a mixture of dietary phenolic compounds exerts a protective effect against inflammation and oxidative stress in the population. These results suggest that cooked ham functionalized with appropriate dietary antioxidants could result in a potential decrease in the risk of certain noncommunicable chronic diseases such as CVD. Future research should explore how polyphenols exert their activity on different metabolic targets, thereby allowing us to observe the absorption, metabolism, distribution, and excretion of these compounds in the diet through marked phenolic compounds.

## Figures and Tables

**Table 1:** Nutritional composition of the cooked ham.

	Control Cooked Ham	Antioxidant Cooked Ham
Energy value (KJ/Kcal)	383.1/91.5	386.4/92.3
Fats (%)	1.5	1.5
Saturated Fats (%)	0.5	0.5
Monounsaturated Fats (%)	0.75	0.76
Polyunsaturated Fats (%)	0.2	0.19
Carbohydrates (%)	1.5	1.46
Sugars (%)	1.2	1.17
Proteins (%)	19.00	19.04
Salt (%)	1.4	1.41
Sodium (ppm)	5650	5523
Selenium (µg/100 g)	6.1	11.00
Zinc (mg/100 g)	0.89	2.9
Vitamin C (mg/100 g)	72.6	124.0
Total Polyphenols (mg/100 g)	0.00	33.69
Chlorogenic Acids (mg/100 g)	0.00	22.50
Catechins and Epigallocatechin (mg/100 g)	0.00	6.58
Hydroxytyrosol (mg/100 g)	0.00	4.45
Other Phenolic Acids (mg/100 g)	0.00	0.16

**Table 2:** Demographic data: means and standard deviations (mean ± SD).

	Control Cooked Ham	Antioxidant Cooked Ham	*p*-Value
Age (years)	40.2 ± 8.3	41.6 ± 9.8	0.540
Men	15	17	0.708
Women	17	16	
Weight (kg)	75.2 ± 13.4	75.2 ± 13.4	0.993
BMI (kg/m^2^)	26.3 ± 3.2	25.1 ± 3.6	0.161
Fat Mass (%)	21.7 ± 7.5	20.8 ± 7.4	0.626
Fat-Free mass (kg)	53.5 ± 11.3	54.4 ± 10.9	0.736
Total Cholesterol (mg/dL)	222.8 ± 36.7	219 ± 30.8	0.645
LDL Cholesterol (mg/dL)	140.9 ± 32.6	137.3 ± 30.8	0.643
SBP (mmHg)	113.6 ±11.2	112.8 ± 11.2	0.789
DBP (mmHg)	71.7 ± 6.8	72.3 ± 9.2	0.784

**Table 3:** Means and standard deviations of the study variables before and after intervention (mean ± SD).

		BASELINE	FINAL	*p*-Value Time	*p*-Value Product × Time
**Laboratory Test**					
Total Cholesterol (mg/dL)	Placebo	222.8 ± 36.7	220.6 ± 38.7	0.528	0.282
	Extract	219 ± 30.8	211.3 ± 33.7	**0.032**	
LDL Cholesterol (mg/dL)	Placebo	140.9 ± 32.6	140.2 ± 32.6	0.857	0.495
	Extract	137.3 ± 30.8	132.7 ± 27.3	0.250	
HDL Cholesterol (mg/dL)	Placebo	63.6 ± 16.5	61.7 ± 15.0	0.859	0.788
	Extract	63.0 ± 18.6	59.6 ± 17.1	0.495	
Triglycerides (mg/dL)	Placebo	91.7 ± 42.8	93.2 ± 45.4	0.859	0.896
	Extract	92.9 ± 48.1	94.8 ± 49.7	0.865	
Oxidized LDL (pg/mL)	Placebo	417.4 ± 273.2	458.0 ± 247.3	0.307	**0.036**
	Extract	443.3 ± 277.6	364.2 ± 212.9	**0.050**	
Oxidized LDL (pg/mL) IMC < 25	Placebo	410.7 ± 231.9	411.5 ± 222.1	0.988	0.638
	Extract	382.3 ± 276.7	348.6 ± 226.1	0.488	
Oxidized LDL (pg/mL) IMC > 25	Placebo	421.9 ± 304.2	489.8 ± 246.8	0.228	**0.026**
	Extract	501.2 ± 266.2	378.6 ± 196.4	**0.050**	
Hs-CRP (mg/L)	Placebo	1.84 ± 1.66	2.03 ± 1.6	0.443	**0.023**
	Extract	2.07 ± 2.37	1.4 ± 1.26	**0.006**	
IL-6 (pg/mL)	Placebo	1.23 ± 1.36	1.20 ± 2.38	0.059	0.331
	Extract	1.37 ± 1.22	0.71 ± 0.47	**0.001**	
MDA (ng/mL)	Placebo	414.6 ± 378.2	434.6 ± 414.5	0.293	**0.035**
	Extract	443.7 ± 294.5	406.6 ± 288.2	**0.050**	
SOD (ng/mL)	Placebo	14.7 ± 3.9	14.6 ± 4.5	0.909	0.247
	Extract	14.8 ± 3.4	13.7 ± 5.5	0.082	
**Blood Pressure**					
SBP (mmHg)	Placebo	113.6 ± 11.2	113.8 ± 11.4	0.953	0.650
	Extract	112.8 ± 11.2	110.3 ± 22.2	0.635	
DBP (mmHg)	Placebo	71.7 ± 6.8	71.4 ± 6.8	0.618	0.538
	Extract	72.3 ± 9.2	71.3 ± 8.8	0.169	
Pulse Pressure (mmHg)	Placebo	41.8 ± 6.5	42.4 ± 6.7	0.276	0.536
	Extract	40.5 ± 6.3	38.9 ± 1.3	0.141	
Daytime SBP (mmHg)	Placebo	116.8 ± 11.5	117.1 ± 12.0	0.735	0.913
	Extract	115.9 ± 12.0	116.4 ± 12.5	0.623	
Daytime DBP (mmHg)	Placebo	74.8 ± 7.5	74.2 ± 7.4	0.530	0.787
	Extract	74.6 ± 8.1	74.2 ± 8.7	0.950	
Daytime Pulse Pressure (mmHg)	Placebo	41.9 ± 6.4	41.9 ± 7.1	0.245	0.916
	Extract	41.3 ± 7.4	42.2 ± 6.7	0.786	
Nighttime SBP (mmHg)	Placebo	107.6 ± 12.4	107.9 ± 12.4	0.799	0.125
	Extract	107.8 ± 10.3	105.7 ± 10.5	**0.050**	
Nighttime DBP (mmHg)	Placebo	65.9 ± 7.0	65.3 ± 6.8	0.462	0.911
	Extract	66.5 ± 9.5	65.7 ± 8.8	0.365	
Nighttime Pulse Pressure (mmHg)	Placebo	41.7 ± 7.6	41.4 ± 7.3	0.341	0.092
	Extract	42.6 ± 8.3	40.0 ± 7.5	0.148	
SBP Load (mmHg)	Placebo	14.1 ± 20.2	13.8 ± 18.6	0.070	0.756
	Extract	14.4 ± 23.3	12.4 ± 23.0	0.247	
DBP Load (mmHg)	Placebo	22.3 ± 20.7	20.1 ± 20.1	0.234	0.478
	Extract	21.1 ± 25.7	20.3 ± 24.6	0.839	
SBP Dipping (%)	Placebo	7.8 ± 6.2	6.7 ± 6.9	0.967	0.163
	Extract	6.7 ± 6.9	8.9 ± 5.5	0.053	
DBP Dipping (%)	Placebo	11.7 ± 7.2	11.8 ± 7.3	0.929	0.830
	Extract	10.9 ± 7.5	11.4 ± 6.0	0.691	

*p* value (time) intragroups (Bonferroni) is reported. *p*-value between-groups in ANOVA for repeated measures with two study factors (Product × Time) is reported. Significant values are in bold.

**Table 4:** Means and standard deviations of the body composition parameters before and after intervention (mean ± SD).

		BASELINE	FINAL	*p*-Value Product × Time
**Body Composition**				
Weight (kg)	Placebo	72.5 ± 13.4	75.0 ± 12.9	0.987
	Extract	75.2 ± 13.4	75.0 ± 13.1	
BMI (kg/m^2^)	Placebo	26.3 ± 3.2	26.2 ± 3.0	0.958
	Extract	25.1 ± 3.6	25.1 ± 3.4	
Fat Mass (%)	Placebo	21.7 ± 7.5	22.0 ± 7.3	0.349
	Extract	20.8 ± 7.4	20.7 ± 7.2	
Fat-Free Mass (Kg)	Placebo	53.5 ± 11.3	53.0 ± 11.0	0.232
	Extract	54.4 ± 10.9	54.3 ± 10.9	

*p* value (time) intragroups (Bonferroni) is reported.

**Table 5:** Systolic blood pressure and diastolic blood pressure dipping data.

SBP Dipping Data				TIME		TOTAL
				BASALINE	FINAL	
Placebo	SBP Dipping	Riser	Count	4	7	11
			% within time	12.5	21.9	17.2
		Non-Dipper	Count	16	15	31
			% within time	50.0	46.9	48.4
		Dipper	Count	12	9	21
			% within time	37.5	28.1	32.8
		Extreme Dipper	Count	0	1	1
			% within time	0.0	3.1	1.6
	Total		Count	32	32	64
			% within time	100.0	100.0	100.0
Extract	SBP Dipping	Riser	Count	5	3	8
			% within time	15.2	9.1	12.1
		Non-Dipper	Count	18	16	34
			% within time	54.5	48.5	51.5
		Dipper	Count	10	13	23
			% within time	30.3	39.4	34.8
		Extreme Dipper	Count	0	1	1
			% within time	0.0	3.0	1.5
	Total		Count	33	33	66
			% within time	100.0	100.0	100.0
**DBP Dipping Data**				**TIME**		**TOTAL**
				**BASALINE**	**FINAL**	
Placebo	DBP Dipping	Riser	Count	3	2	5
			% within time	9.4	6.3	7.8
		Non-Dipper	Count	9	12	21
			% within time	28.1	37.5	32.8
		Dipper	Count	17	14	31
			% within time	53.1	43.8	48.4
		Extreme Dipper	Count	3	4	7
			% within time	9.4	12.5	10.9
	Total		Count	32	32	64
			% within time	100.0	100.0	100.0
Extract	DBP Dipping	Riser	Count	3	0	3
			% within time	9.1	0.0	4.5
		Non-Dipper	Count	12	16	28
			% within time	36.4	48.5	42.4
		Dipper	Count	16	14	30
			% within time	48.5	42.4	45.5
		Extreme Dipper	Count	2	3	5
			% within time	6.1	9.1	7.6
	Total		Count	33	33	66
			% within time	100.0	100.0	100.0

## Data Availability

No new data were created or analyzed in this study. Data sharing is not applicable to this article.
